# Stereo Camera Based Virtual Cane System with Identifiable Distance Tactile Feedback for the Blind

**DOI:** 10.3390/s140610412

**Published:** 2014-06-13

**Authors:** Donghun Kim, Kwangtaek Kim, Sangyoun Lee

**Affiliations:** 1 School of Electrical and Computer Engineering, Purdue University, West Lafayette, IN 47906, USA; E-Mail: zava@purdue.edu; 2 Department of Electrical and Electronic Engineering, Institute of BioMed-IT, Energy-IT and Smart-IT Technology (Best), Yonsei University, Yonsei-ro, Seodaemun-gu, Seoul 120-749, Korea

**Keywords:** finger pointing gestures, 3D pointing direction estimation, obstacle detection, stereo camera system, human computer interaction, tactile feedback, virtual cane

## Abstract

In this paper, we propose a new haptic-assisted virtual cane system operated by a simple finger pointing gesture. The system is developed by two stages: development of visual information delivery assistant (VIDA) with a stereo camera and adding a tactile feedback interface with dual actuators for guidance and distance feedbacks. In the first stage, user's pointing finger is automatically detected using color and disparity data from stereo images and then a 3D pointing direction of the finger is estimated with its geometric and textural features. Finally, any object within the estimated pointing trajectory in 3D space is detected and the distance is then estimated in real time. For the second stage, identifiable tactile signals are designed through a series of identification experiments, and an identifiable tactile feedback interface is developed and integrated into the VIDA system. Our approach differs in that navigation guidance is provided by a simple finger pointing gesture and tactile distance feedbacks are perfectly identifiable to the blind.

## 1. Introduction

The role of the white cane, a mechanical device, is an extended-hand like guidance for the better mobility that guarantees safe and comfortable movements. It is helpful for the blind to avoid obstacles and to negotiate his/her steps, and to follow the safest walking trajectory while in motion. Over decades, the stick-look cane has been recognized as a commonly used tool for people who are blind or visually impaired as compared to guide dogs that cost more. Despite the popularity of the white cane, it has drawbacks such as a long training time, a limited sensible range (e.g., only usable within 1–2 m), uncomfortable carrying and contact-based object detection.

From a technical point of view, the virtual cane system can be separated into two main parts: sensing obstacles and providing feedbacks to avoid the detected obstacles. Literally, sensors play a crucial role in sensing obstacles and these days high-tech sensors like ultrasound and lidar have been used as new approaches [1–3]. Nonetheless, those sensors have tradeoffs in terms of accuracy, cost and portableness, and so camera sensors have been considered as the best option due to the unique benefits such as low cost, non-contact object detection, precise shape reconstruction, and computational efficiency. These benefits have been also proved by researchers [4,5] who demonstrated that utilizing vision sensors benefit the blind to explore visual environments efficiently under dynamic scenes in various applications.

Recently, stereo camera based approaches have been introduced by several researchers. Jose *et al.* [6] developed a virtual cane system by using a stereo camera, and successfully showed the effectiveness as a wearable system customized for assisting navigation under unknown environments. Fernandes *et al.* [7] proposed a robust stereo vision algorithm extracting predefined landmarks like circles that provide cues for safe walking. As a hybrid system, Brilhault *et al.* [8] combined stereo vision and a GPS system to improve user's positioning. An assistance system that can guide user's orientation to locate objects has been developed by Dramas *et al.* [5].

Denis *et al.* [9] developed a wearable virtual cane system that can detect objects coming close to the user. Additionally, they designed distinct sound feedbacks for the estimated distance to the detected objects. Although many interesting systems have been developed until now, most of the systems are passive or workable only under some conditions like known environments and predefined landmarks. These limitations could be barriers to design a natural user interface.

For a natural user interface, vision based hand gesture and finger pointing technologies have been actively developed by many researchers [10–14] since those are non-intrusive, convenient, and interactive. Especially, 3D range data based gesture recognition is highly reliable and robust to be a practical use as demonstrated in many game applications with the Kinect. The effectiveness of using 3D depth images for finger gesture recognition has also been corroborated by Matikainen *et al.* and Nickel *et al.* [13,15] who developed robust pointing gesture technologies for interactive visual scene analyses. These pointing gesture technologies provide users with benefits in that the user's hand can become free from holding a sensing device and the user is interactively able to get accurate information of a place where he/she wants to explore in advance.

In general, developing finger pointing recognition technologies is threefold: finger detection and tracking, estimation of finger pointing directions, and obstacle's detection. First, detecting and tracking fingers with wearable cameras is not simple due to human motions and noisy backgrounds. As pioneering work, several researchers [16–18] introduced hand detection and tracking algorithms with simplified conditions like uniform backgrounds or wearing color gloves. Afterward, many researchers put a lot of effort into improving bare hand tracking technologies on cluttered backgrounds for sign language applications [19,20] and for human computer interactions [21]. In our work, a dynamic update model for moving backgrounds was proposed to compensate background changes caused by motions from body worn cameras.

Second, with the detected pointing finger, estimating an accurate 3D finger pointing direction can be achieved by using classic theories in stereo vision [22], multiple view geometry [23], and stereo camera calibration [24,25]. In our study, we utilized the existing theory to estimate a 3D pointing direction with disparity data obtained from a stereo camera. Compared with the previous steps, detecting obstacles is a challenging problem since algorithms should be able to detect obstacles even in complex surroundings as humans naturally do. To detect objects accurately, segmentations that intelligently extract target objects under dynamic scenes are extremely important. As a new algorithm, we developed a robust segmentation algorithm which is suitable for the virtual cane system.

In order to deliver the visual scene information to the blind user, providing feedbacks via sounds and/or vibration is effective. However, audible feedback often becomes noise or even can mask important information such as traffic sounds and other people's talk on street. For this reason, tactile feedback is preferably developed as a non-intrusive interface with vision systems. As the first work, Pressey [26] developed a lightweight hand-held device called MOWAT SENSOR to be easily carried while walking. The sensor itself detected an object within a beam of high frequency sounds and vibrated for tactile feedback with predefined vibrations (e.g., the higher frequency is interpreted as a closer obstacle). Etran *et al.* [27] invented a wearable navigation system based on a haptic directional display embedded in the back of a vest. Directional cues were generated differently like lines, circles and blinking to inform directional cues.

Velazquez *et al.* [28] introduced a concept of Intelligent Glasses with tactile feedback. A dynamic edge shape extracted from a stereo camera was directly transmitted to a tactile display, a braille built with push up/down mechanical sticks representing the edge shape. A shortcoming of the system was that user's hand had to be placed on the haptic display device all the time to feel tactile feedbacks. As recent work, Hirose and Amemiya [29] developed a prototype with a PDA (Personal Digital Assistant) device. For tactile feedback, three vibrating motors attached to the user's arms (left and right) and back. Direction cues were delivered to the user by vibrations on single or double motors, while the number of pulses of the vibrations was used for distance cues. However, none of the existing tactile feedback systems did design identifiable feedback signals based on human perceptions but instead intuitively selected vibration signals. In our work, we designed perfectly identifiable tactile signals by conducting a series of identification experiments and those signals were successfully integrated in our virtual cane system.

In this paper, we focus on presenting a robust and advanced distance estimation system with a stereo camera that is operated by a simple finger pointing gesture. Additionally, we propose a complete virtual cane solution by integrating a tactile feedback interface that employs perceptually identifiable frequencies, obtained from 1D frequency identification experiments for distance-matching tactile feedbacks.

The remainder of this paper is organized as follows. In Section 2, we describe the visual information delivery system, and the experimental results are presented in Section 3. Section 4 explains how we designed identifiable tactile feedback signals and integrated the tactile feedback system with the visual information delivery system. Conclusions and future work are provided in Section 5.

## 2. Visual Information Delivery Assistant (VIDA)

**Figure 1.**
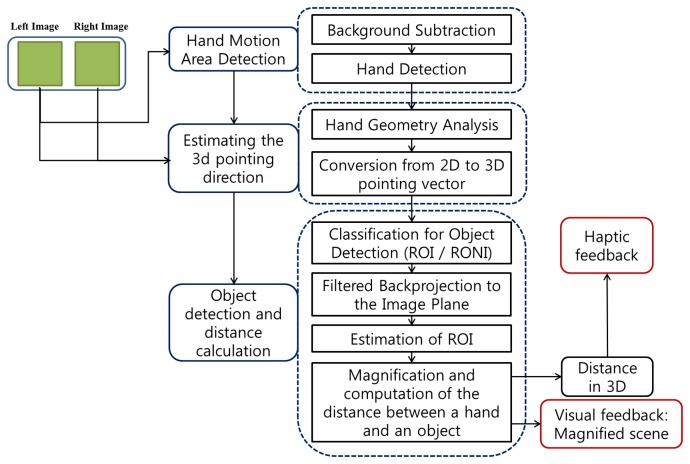
The flow chart of algorithms used in Visual Information Delivery Assistant.

The Visual Information Delivery Assistant (VIDA) consists of three steps: hand detection, estimation of a 3D pointing direction, object detection and distance calculation. The flow chart of the algorithm is shown in Figure 1.

### 2.1. Hand Detection

As earlier mentioned, extracting hands or a finger in a complex scene is not easy. The problem even becomes severe if images are taken from a fluctuated camera, which is the case of wearable virtual cane systems. To tackle the challenging problem, we combine static and dynamic segmentation methods to improve hand region detection. Hand and finger regions are then detected by using skin color information. In the following, detailed algorithms are explained.

#### 2.1.1. Background Subtraction under Dynamic Scenes

Background subtraction provides a fundamental framework for hand area detection in both static and dynamic environments. In our algorithm, we made a fusion framework that adaptively utilizes static and dynamic background subtraction methods. For this approach, we define a static background as an image frame whose variations are relatively small, while a dynamic background is an image frame that has big global variations (e.g., the entire scene is changed). To implement the static background subtraction, we adopted a learning average method, a well-known statistical method, to build a background model. The method basically creates a background model based on the mean difference computed from a set of accumulated image frames. Then, subsequent frames are subtracted from the created background model, which results in segmentation. This simple approach enables the quick detection of a moving hand in a static environment.

For taking advantage of the static background subtraction approach in dynamic scenes, we introduce a strategy that makes our system intelligently works well under dynamic environments. The strategy includes continuous dynamic model updates and detecting global scene changes to trigger building a new background model. The former develops an extended version of static background subtraction by replacing the static background model with the most up-to-date background model that is captured from dynamic scenes. In other words, any dynamic scene captured at time *t* can be considered to be a new static background model for hand segmentation under dynamic environments. This approach works well for both static and dynamic background subtraction.

**Figure 2.**
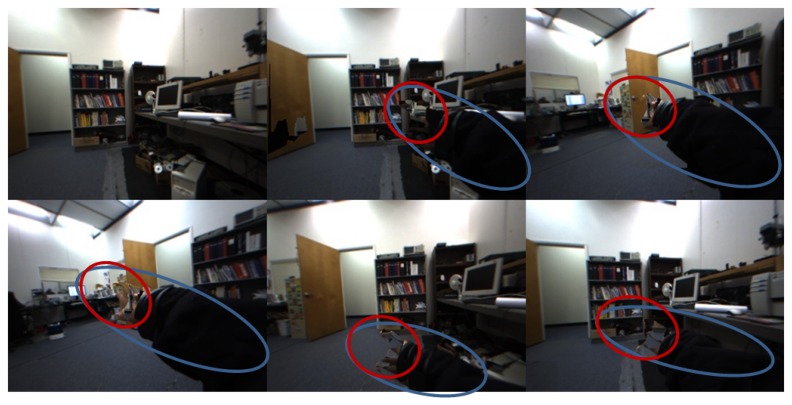
Examples of hand detection with our background subtraction under dynamic scenes: a pointing finger (red) and a moving arm (blue). Note that images were taken sequentially from left to right.

However, one issue here is how to let our system know the update timing for a new background model when the stereo camera is in motion. In our observation, two typical types of global changes were found in a dynamic environment: changes by the moving camera and local changes by moving objects. The latter must be excluded for updating the model. This is why our strategy includes detecting global scene changes. For this, we developed a decision maker algorithm that compares local variations within predefined window blocks to determine whether the global scene was changed or not. A threshold value was defined by our pilot study for the criterion. The sum of local variations is compared with the threshold. For instance, the greater value is the case of global scene changes that result in creating a new background model, while the opposite case updates the background model locally from the previous model. During this process, the hand region is not updated. Figure 2 shows how the background model is updated against camera motions and a moving object (the user's arm).

For efficient processing, the input image (320 × 240 pixels) was divided into 8 by 6 blocks (40 × 40 pixels). For each block, our decision maker algorithm was run to determine whether a new global background model should be created. If more than half pixels in each block show variations, the block is categorized as a block to be updated. The background model is then updated with the eight nearest neighbors of the block. In our approach, both a color background model and a depth background model are updated except the foreground region of a skin-colored hand area for the next process.

#### 2.1.2. Hand Detection Using Color Segmentation

With the detected region including the user's arm, the hand region is identified by skin-color information. Two color spaces, *YUV* and *YIQ*, are used together for creating a unique classifier for color segmentation in our approach. Our classifier was designed to take two feature parameters from the two color spaces (*i.e.*, one from each). The reason why we have chosen the specific color models is that both models are sensitive to low color depths (image bandwidth) like human visual perception. It is also known that *YUV* and *YIQ* have the same luminance component but different coordinate systems for chrominance components. For generating feature parameters with the *YUV* model, luminance (*Y*) and two chrominance components (U and *V*) are computed from the *RGB* space by the transformation matrix below:

(1) $$\left[\begin{array}{c} Y\\ U\\ V \end{array}\right]=\left[\begin{array}{ccc} 0.299 & 0.587 & 0.114\\ -0.147 & -0.289 & 0.436\\ 0.615 & -0.515 & -0.100 \end{array}\right]\;\left[\begin{array}{c} R\\ G\\ B \end{array}\right]$$

The computed *U* and *V* values are then used for computing two feature parameters, a displacement vector (*C*) and the phase angle (*θ*), that can be used for color segmentation. Those features are computed as follows:

(2) $$C=\sqrt{{\left|U\right|}^{2}+{\left|V\right|}^{2}}\;\text{and}\;\theta =\tan^{-1}(V/U)$$

In the *YIQ* color space, *I* and *Q*, representing chrominance values, can also be used as features. Obtaining these values from *RGB* is achieved by the transformation matrix:

(3) $$\left[\begin{array}{c} Y\\ I\\ Q \end{array}\right]=\left[\begin{array}{ccc} 0.299 & 0.587 & 0.114\\ 0.596 & -0.274 & -0.322\\ -0.212 & -0.523 & 0.311 \end{array}\right]\;\left[\begin{array}{c} R\\ G\\ B \end{array}\right]$$

**Figure 3.**
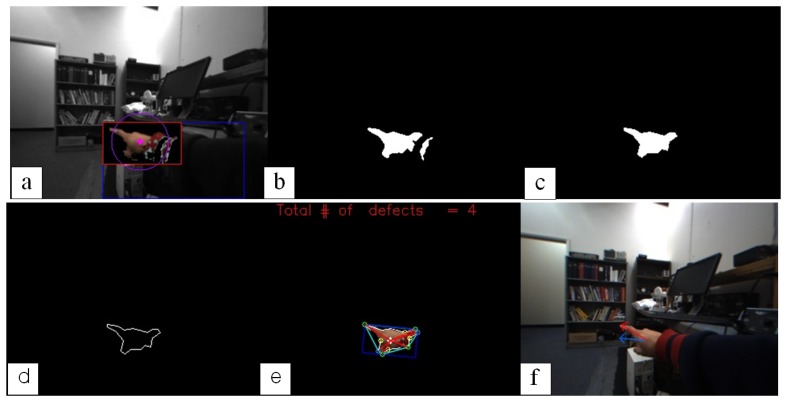
Photographic representation of estimating a 2D pointing direction: **(a)** a detected hand area; **(b)** the binary image of **(a)** but with noises; **(c)** a cleaned hand region after removing noises by using a connected component analysis; **(d)** the hand contour; **(e)** a hand shape estimated by an geometric analysis; and **(f)** an initial estimated direction vector (blue) by a blue bounding box and a refined direction vector (red) by a hand shape geometry.

A combination of the four feature values, *C*, *θ*, *I* and *Q*, can create a unique criterion that can accurately segment human hands. Therefore those values can be tuned up to the aiming level of segmentation. In our application, we used only two features, *θ* and *I*. The ranges of optimized values used for hand segmentation are 105 to 150 and 15 to 100, respectively. After detecting the skin colored area with these color features, noises were completely eliminated by using a connected component analysis. The upper row of Figure 3 shows this segmentation procedure using the two feature values.

### 2.2. Estimation of 3D Pointing Direction

A pointing gesture with fingers generally forms a particular hand shape created by a combination of convex and concave shapes. We utilize this phenomenon to estimate the finger pointing direction in 2D space based on a shape analysis, and the estimated direction is then extended to three dimensions using the existing camera geometry We further present these two steps in the following.

A pointing direction in 2D space is estimated by taking three steps, as seen in the lower row of Figure 3: extracting the hand contour, finding a convex polygon on the extracted contour, and estimating an accurate pointing vector. In order to extract a precise hand contour, the polygonal approximation is applied because of its robustness to illuminations. The extracted contour is then verified by a convex hull algorithm that finds both convex vertex points and convex defects (concave). The process of examining convexity is as follows:

Considering two points, *A* and *B* in a region Ω, the convexity can be evaluated by the following measure:

(4) V=αA+βB

for 0 < *α, β* < 1 and *α* + *β* = 1.

If all possible *V* are in the region Ω for arbitrary values of *α* and *β*, then the contour from *A* to *B* is convex. This way, a convex polygon and convexity defects are generated as the output.

As the last step, an accurate 2D pointing direction is estimated by taking two steps, an initial estimate and the refinement process, with the filtered hand contour (Figure 3d) and a hand shape polygon (cyan colored lines in Figure 3e) formed from finger tips and convexity. An initial direction is roughly determined from a bounding rectangle of the hand contour, a blue box in Figure 3e. That is, the longer side of the rectangle becomes a unit vector, a blue arrow in Figure 3f, of the 2D pointing direction at the geometric center of the hand. The initial pointing direction is then refined towards the index finger by the principle vector of the hand shape polygon (see the longer red line inside the hand shape contour in Figure 3e). The finally estimated 2D pointing direction is visualized as a red arrow superimposed on the hand image in Figure 3f.

**Figure 4.**
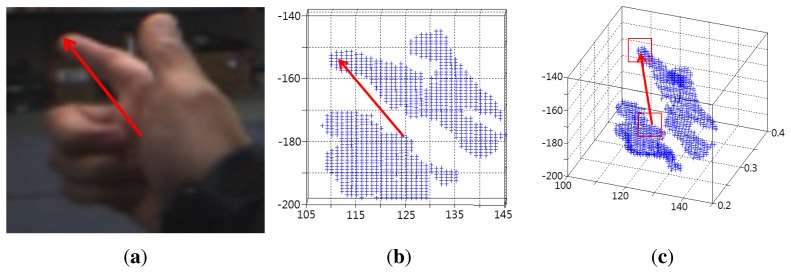
Estimation of a 3D pointing direction: **(a)** a 2D pointing vector; **(b)** a hand area projected onto the *x-y* plane; and **(c)** an estimated 3D vector from the corresponding 2D pointing vector.

To estimate the corresponding 3D pointing vector, intrinsic parameters computed from the camera calibration are used. In theory, 3D points on a 3D pointing vector from the corresponding 2D points are obtained as follows:

(5) $$Z=\frac{f}{d}B,\;X=\frac{u}{f}Z,\;Y=\frac{v}{f}Z$$

where a 3D point is denoted by a vector (*X*, *Y*, *Z*) from the origin in the camera coordinates, f is the focal length of the camera, B is the baseline distance of a stereo camera, *d* is the disparity value at any location, and (*u*, *v*) is a location on the 2D image. Figure 4 graphically shows how a 3D pointing direction is estimated from a 2D pointing direction.

### 2.3. Object Detection

In the VIDA system, any object inside Region of Interest (ROI) extracted by user's pointing gesture is detected as an obstacle. The actual distance to the detected object is accurately computed with the stereo camera geometry that provides transformation between 2D and 3D spaces. In the following, the specific algorithms, classification of 3D points and ROI extraction, are presented.

#### 2.3.1. Classification of 3D Points

Given a 3D pointing vector estimated in the previous step, a line passing through any two points in 3D space can be simply obtained in homogeneous coordinates [23]. For simplicity's sake, we do classify 3D points of object candidates in 2D image planes (*x-z* and *y-z* projected planes) instead of 3D space. By doing so, the computational cost, required for distance computing between 3D points and a 3D line (e.g., a finger pointing vector), has been greatly reduced. The classification algorithm therefore works as follows. First, the line (*l*) between two 2D points (*p*_1_, *p*_2_) as projected to a 2D plane is computed by the cross product of the two points,

(6) $$l=p_{1}\times p_{2}$$

where *l*, *p*_1_, *p*_2_ ∈ *R*^3^ are represented in the homogeneous coordinates.

Second, using Equation (5), all pixels in a 2D image are mapped to the corresponding 3D points by the stereo camera geometry (or called two view geometry). Each 3D point is then classified into two groups (class of interest (*C_I_*) and class of non-interest (*C_NI_*)) by the measured distance in the *x-z* and *y-z* image planes. The orthogonal distance from a point to a line is obtained by the dot product:

(7) l·p=d

where *l*, *p* ∈ *R*^3^ in the homogeneous coordinates and d is a scalar.

**Figure 5.**
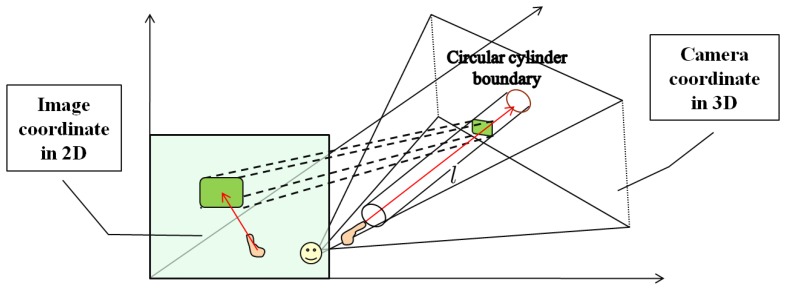
Relationship between the image coordinates and the camera coordinates.

With a fixed value of *d* (orthogonal distance), a virtual cylinder is formed as shown in Figure 5 and its boundary, defined by *d*, becomes the classifier of 3D points. Based on this configuration, any 3D point inside the virtual cylinder falls into the class of interest (*C_I_*), and otherwise belongs to the class of non-interest (*C_NI_*). Then, all points in *C_I_* are back-projected onto the corresponding 2D image plane, and the region becomes a ROI candidate for object detection. This transformation between 2D image coordinates and 3D camera coordinates is illustrated in Figure 5. Note that in the figure a smile face pictogram represents the center of the camera coordinate system.

#### 2.3.2. ROI Extraction and Object Detection

**Figure 6.**
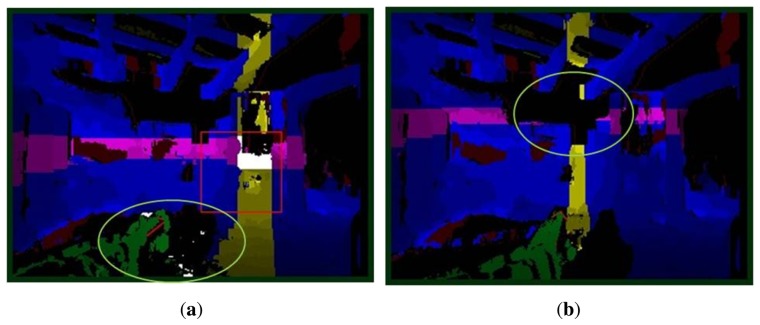
Examples of **(a)** noisy disparity data and **(b)** lack of disparity values. Those areas are highlighted by ellipsoids.

As outcomes in the previous step, ROI candidates were generated on the image plane from 3D points. However, it was observed that ROI candidates are not correctly generated in case of disparity errors (noise or lack of data) as seen in Figure 6. These problems are mainly caused by illumination changes and errors in computing disparity values. To fix the problems, 3D disparity noises are filtered first, and then the way of classification of 3D points is slightly modified from the previous.

**Figure 7.**
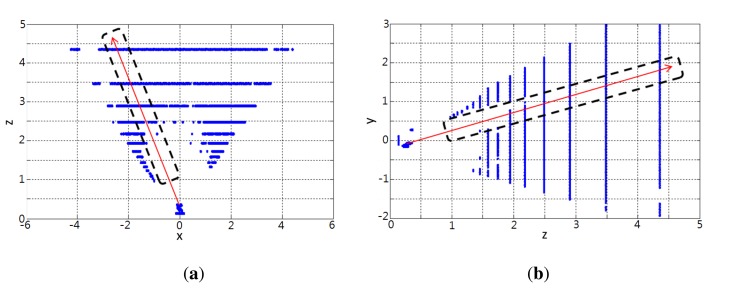
Virtual rectangles used as a classifier on **(a)** the *x-z* plane and **(b)** the *y-z* plane.

**Figure 8.**
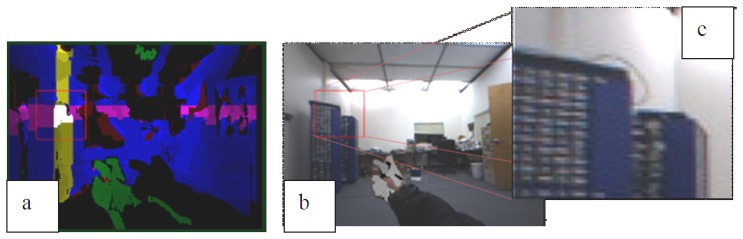
ROI extraction and the magnified view: **(a)** the extracted ROI region highlighted by a red rectangle; **(b)** Superimposed ROI; and **(c)** its magnified view.

As seen in Figure 7, 3D points are classified by using two virtual rectangles projected from the virtual cylinder onto the x-z and y-z planes respectively. In last to extract the final ROI candidate, the classified 3D points in the ROI interest group are projected onto the image plane in which two perpendicular bars are formed. The intersection of the two bars (yellow and pink) shown in Figure 8 is chosen as the final ROI. The white region in Figure 8a is the ROI candidate determined by the virtual cylinder only.

In our VIDA system, the final ROI can be visually magnified for the user to perceive the details (texture information) of the detected objects. The magnification of the ROI is performed using the bilinear interpolation method. The bilinear interpolation performs a linear interpolation in one direction first and then repeats it for the other direction. The resulting images by the ROI extraction and magnification are shown in Figure 8.

## 3. Experimental Results of VIDA

**Figure 9.**
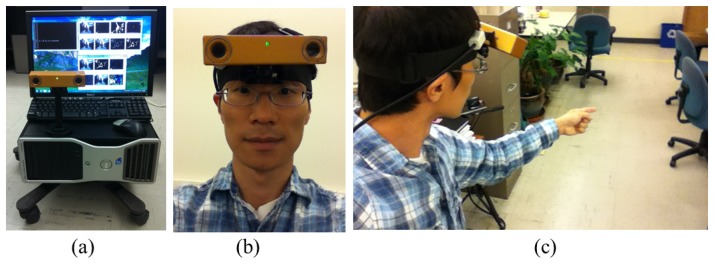
Our developed VIDA system: **(a)** the entire system look; **(b)** an example of the head mounted VIDA system; and **(c)** the user scenario.

The VIDA system shown in Figure 9 consists of a commercial stereo camera (Bumblebee 2 manufactured by PointGrey Inc., Richmond, BC, Canada), a personal computer (Intel Core2 2.2G Hz and 2G RAM) and a standard LCD (Liquid-Crystal Dispaly) monitor. The system runs at 6 frames per second with an input QVGA (Quarter Video Graphics Array, 320 × 240 pixels) image. The spatial accuracy, reported by the manufactured company as the system calibration error, was 5 mm at 1 m distance, which is sufficiently accurate to detect hands and objects for the virtual cane system.

**Figure 10.**
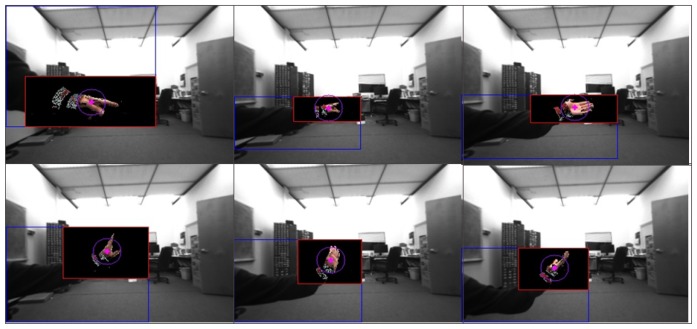
Results of hand detection.

**Figure 11.**
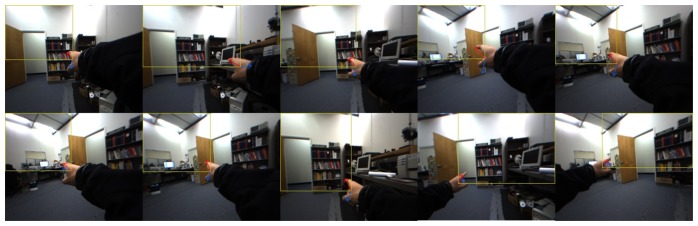
Results of finger pointing estimation (red arrow) with dynamic backgrounds.

Our developed system, VIDA, was thoroughly verified in terms of accuracy and robustness against illuminations and occlusions. The evaluation was done systematically, first with each algorithm and then with the whole system. The results shown in Figure 10 show how our hand detection algorithm works well with backgrounds that contain numerous objects. Figure 11 also demonstrates the precise 3D pointing estimation under a dynamic environment. In the resulting images, the estimated pointing vector colored in red was superimposed onto user's hand to show the accuracy.

**Figure 12.**
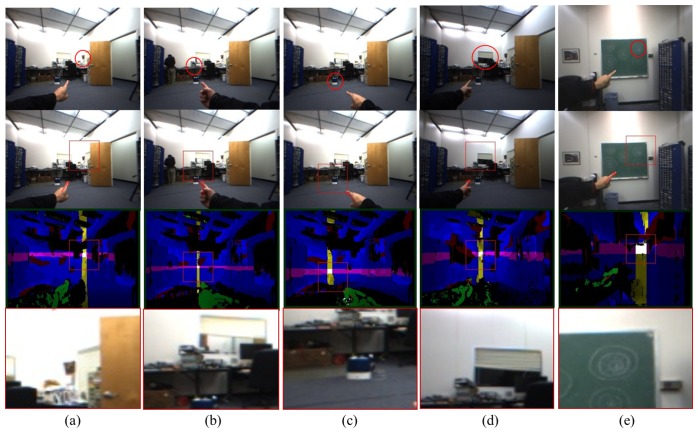
Results of object detection and magnification for **(a)** a calendar; **(b)** an instrument; **(c)** a humidifier; **(d)** a small window; **(e)** a drawn shape on a blackboard. Each row, from top to bottom, shows the sequential order for ROI extractions and the magnified ROI display.

**Figure 13.**
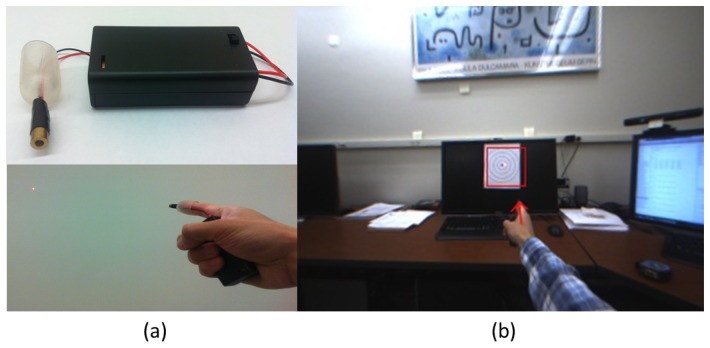
Quantitative experimental setup to evaluate our finger pointing algorithm with a laser pointer (ground truth): **(a)** a laser pointer attached to the top of the index finger and **(b)** the experiment with a target circle image at a distance (1 m).

For the system evaluation, we tested VIDA with various objects that are different in shape, color and size under dynamic scenes. For this experiment, a user was asked to walk around and make a random pointing gesture to objects. To evaluate the accuracy, what was pointed by the user has been recorded and compared with the detected object from VIDA. The sequential images seen in Figure 12 demonstrate the experiment procedure. As clearly seen in Figure 12, all objects pointed by the user were successfully detected even at long distance (up to 3 m). The numerical results are summarized in Table 1. For a more accurate evaluation with the ground truth, we used a laser pointer, attached to the top of the index finger, as a tool that can generate the ground truth. Figure 13 shows the experimental setup and the image of pointing target circles. During the experiment, points marked by laser points on the target image were recorded and compared with pointing points estimated by our algorithm. Geometric errors between the laser points (ground truth) and the estimated points were computed and averaged for 40 repetitions per distance, 1, 2 and 3 m, respectively. The results were also summarized in Table 2.

**Figure 14.**
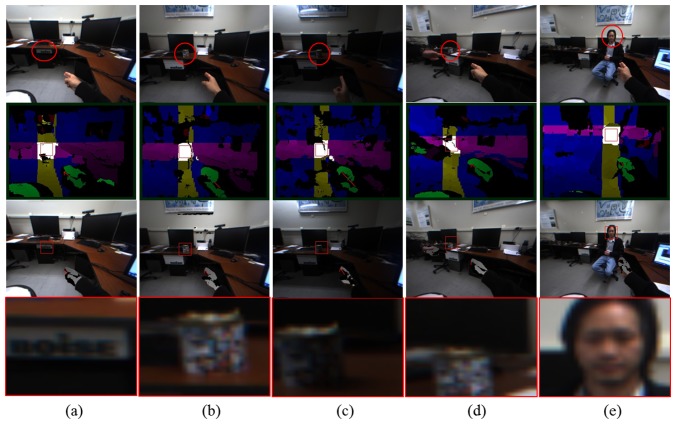
Results with varying illumination conditions and occlusions: **(a**–**c)** three lighting conditions (bright, normal, dark) and **(d,e)** occlusions by other people's hand and face.

Additionally, robustness against illuminations and occlusions has been tested. For the illumination test, three different lighting conditions (bright, normal, dark) were used and compared with each other in setting that these lighting conditions are distinguishable on the taken images. For the evaluation with occlusions, two user scenarios were developed: interferences by extra hands and faces since both cases can significantly affect the performance of our system utilizing skin color information for hand detection. The results in Figure 14 show the robustness of our system against illuminations and occlusions.

## 4. Development of Tactile Feedback Interface for VIDA

In this section, we first describe a frequency identification experiment conducted to choose a set of distinctive tactile signals. We then present a tactile feedback interface integrated into VIDA.

### 4.1. Finding Identifiable Frequencies for Tactile Feedback

We designed an identification experiment to find a set of identifiable signals for tactile feedback. In the design of haptic feedback signal waves, we focused on frequency identification because our hardware setup with a mini piezo driver (DRV8662, manufactured by Texas Instrument Inc., Dallas, TX, USA) provides the wider range of responses in frequency than in amplitude. For the experimental setup, cycle and amplitude were fixed to 3, 60 Vpp, and a square wave, based on user's preference. Additionally, a square waveform was selected because it delivers the stronger haptic effect than other wave forms (sinusoidal or sawtooth) when the same voltage input is given. We were particularly interested in finding identifiable frequencies on the index finger since the goal of the present study is to develop a virtual cane system that can be operated by a simple pointing gesture. It is also well known that the index finger is most sensitive to tactile sensation in our body [30].

**Figure 15.**
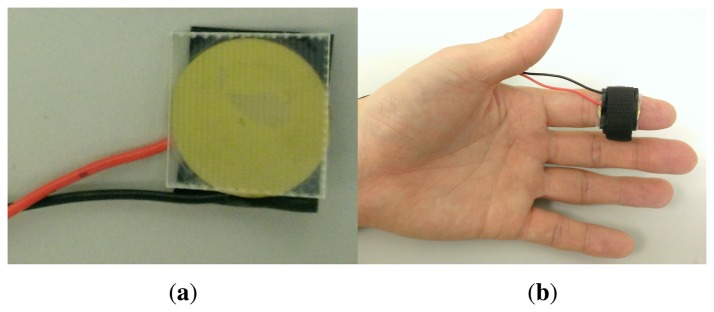
Identification experiment setup to investigate identifiable frequencies for tactile feedback: **(a)** Piezoelectric actuator and **(b)** the actuator attached to the index.

For the identification experiment, a vibrator (see Figure 15a) for tactile feedback was built with a piezoelectric actuator (20 mm diameter, Murata Manufacturing Co. Ltd., Nagaokakyo, Kyoto, Japan), and was affixed to a transparent acrylic square cut (20 mm long and 2 mm thick). A programmable piezo actuator driver (DRV 8662 EVM, Texas Instrument Inc.) was used to drive the vibrator. Tactile signals were pregenerated with square waves at different frequencies and then sent to the piezo driver automatically, whenever the participant press a key to feel the next test signal. Ten participants (4 females and 6 males; age range 22–36; no previous haptic experience; neither visually impaired nor blind) took part in the identification experiment.

In the experiment, all participants were instructed to put the vibrator on their index finger and took five minute training to get familiar with tactile sensations at different frequencies to be tested. Vibrations with the three frequencies were randomized and presented to each participant one by one at a time. The participant then had to respond immediately the identification number of the presented frequency by using the keyboard. A PC (Personal Computer) monitor graphically displayed all necessary information (questions, trials remained, the elapsed time) for the participant to follow the procedure until complete the experiment. In order to obtain unbiased data, a minimum of 50 trials each frequency as suggested by Miller [31], that makes a minimum of 150 trials in total, was tested for an identification experiment.

The experiment was repeated three times with different sets of frequencies as shown in Table 3 with the same participants. The very first experiment tested whether an initial set of three frequencies (10 Hz, 100 Hz and 300 Hz) are identifiable, and the second experiment was then conducted with a new set of frequencies (10 Hz, 100 Hz and 500 Hz) that was formed by replacing 300 Hz with 500 Hz after investigating the result of the first experiment. In the last experiment, a new frequency, 600 Hz, was verified instead of 500 Hz because the frequency 500 Hz was not perfectly identified. In this way, a final set of identifiable frequencies (10 Hz, 100 Hz and 600 Hz) was found. Table 3 shows the three confusion matrices obtained through the three consecutive identification experiments. All of the participants completed each experiment in 40 min, and so it took about two hours in total for each participant to complete the entire experiment including a 10-min break.

### 4.2. Design of Tactile Feedback Interface with Identifiable Frequencies

**Figure 16.**
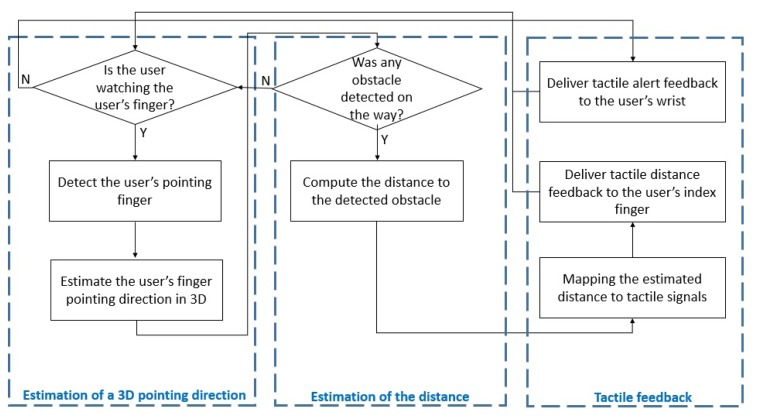
A flow chart of a complete solution of our proposed virtual cane system including a tactile feedback interface.

With the result of the frequency identification experiment, we propose a novel tactile feedback interface that can be integrated into the VIDA system. Our design takes into account both identifiable distance feedback and hand guidance feedback keeping user's hand to be in the camera's view of VIDA. For the tactile distance feedback, the distance estimated from the VIDA system is mapped to one of the three identifiable frequencies (10 Hz, 100 Hz and 600 Hz) in Table 4. For instance, the higher frequency is assigned to the closer distance since users have to take a quicker action to avoid detected obstacles. On the contrary, the guidance feedback uses the highest frequency (600 Hz) since it is provided only when user's hand is out of the camera view and in general, people perceive signals at higher frequencies as warning signals. The two signals are delivered to two separate haptic actuators, attached to the index finger for the distance feedback and the wrist for the guidance feedback as an example. The interpretations are summarized in Table 5. Figure 16 shows how the designed tactile feedback interface can be successfully integrated into the VIDA system. The developed tactile feedback interface can also be used for other navigation systems as long as distinctive distance values are provided.

## 5. Conclusions

We developed a complete solution of a virtual cane system by combining finger pointing gesture and tactile feedback. For the development of finger pointing estimation, a novel algorithm that can precisely estimate a 3D finger pointing direction with a stereo camera was proposed. The proposed algorithm was thoroughly tested under various conditions (dynamic scenes, different objects, illumination changes and occlusions). The evaluation results show that our developed system (VIDA) is sufficiently robust and provides accurate object detection. In addition, we designed identifiable tactile signals that can be mapped to distance information estimated by VIDA. Those signals (10 Hz, 100 Hz, 600 Hz) were selected through identification experiments and were then used for developing a tactile feedback interface. As the last step, we have demonstrated that the tactile feedback interface can be successfully integrated into VIDA as a virtual cane system.

Our developed visual system provides accurate finger tracking and finger pointing estimation in real-time. The accuracy and real time performance enable blind people to navigate on street only with a simple finger pointing gesture. This technology is not only a cost effective solution, but is also extendable for other applications such as finger or hand gesture controls for mobile devices, computer games and VR (virtual reality) applications. Towards a complete navigation solution for the blind, we adopted haptic feedback that is an effective way to deliver obstacle's information under dynamic and noisy environments on street. Unlike other prior work [26–28], we adopted identifiable tactile signals that were designed by identification experiments. This approach can benefit researchers or designer who develop human computer interfaces concerning haptic perception. In last, our system differs in that navigation guidance is given upon user's simple gesture action (*i.e.*, both way interactions) and our approach is robust under unknown dynamic scenes.

Our future work will continue to improve the frame update rate (6 Hz) for faster walkers and will also evaluate the proposed virtual cane system by conducting user studies with visually impaired people. We are also interested in learning more about the possibility of designing tactile signals with other parameters (amplitudes and complex wave forms) by conducting more identification and psychophysical experiments.

## Figures and Tables

**Table 1.** The accuracy of object detection from Figure 12.

	**(a)**	**(b)**	**(c)**	**(d)**	**(e)**
Object size	8 × 18	12 × 17	13 × 16	36 × 17	17 × 17
Distance error (pixels)	7.28	8.06	6.40	8.00	7.07

**Table 2.** Quantitative experiment results of our proposed pointing algorithm with using a laser pointer.

**Distance**	**1m**	**2m**	**3m**
Average error (pixels)	8.46	11.27	13.56
Average error (cm)	6.98	15.21	25.58
Standard dev. (cm)	3.43	3.25	6.39

**Table 3.** Stimulus-response confusion matrices obtained through three repetitions of an frequency identification experiment with different sets of frequencies. Each cell shows accumulated responses from ten participants. Note that the max number “500” indicates perfect identification.

	**Response (Experiment 1)**				**Response (Experiment 2)**				**Response (Experiment 3)**			
Stimulus	Hz	10	100	300	Hz	10	100	500	Hz	10	100	600
	10	**500**	0	0	10	**500**	0	0	10	**500**	0	0
	100	0	**431**	69	100	0	**488**	12	100	0	**500**	0
	300	0	60	**440**	500	0	9	**491**	600	0	0	**500**

**Table 4.** A proposed set of identifiable tactile signals with dual actuators used for distinctive distance ranges.

**Actuator I (Distance Feedback)**		**Actuator II (Guidance Feedback)**

Distance (m)	Frequency (Hz)	Frequency (Hz)
*D*<1	600	600
1 ≰ *D* <2	100	
*2* ≰ *D* <3	10	

**Table 5.** A configuration of dual actuators with its interpretations.

**Interpretation**	**Actuator I**	**Actuator II**
Display distance information	ON	OFF
Alert to align watching and pointing	OFF	ON
